# 11-Oxygenated androgens are not secreted by the human ovary: *in-vivo* data from four different cases of hyperandrogenism

**DOI:** 10.1530/EJE-22-0518

**Published:** 2022-10-13

**Authors:** Matthias K Auer, James M Hawley, Christian Lottspeich, Martin Bidlingmaier, Andrea Sappl, Hanna F Nowotny, Lea Tschaidse, Marcus Treitl, Martin Reincke, Brian G Keevil, Nicole Reisch

**Affiliations:** 1 Medizinische Klinik and Poliklinik IV, Klinikum der Universität München, LMU München, Munich, Germany; 2 Department of Clinical Biochemistry, Manchester University Foundation NHS Trust, Manchester Academic Health Sciences Centre, Southmoor Rd, Manchester, UK; 3 Department for Radiology, Neuroradiology and Interventional Radiology, Trauma Centre Murnau, Germany; 4 Clinic and Polyclinic for Radiology, Clinical Centre of the University of Munich, LMU Munich, Germany

## Abstract

**Objective:**

Differentiation of an adrenal from an ovarian source of hyperandrogenemia can be challenging. Recent studies have highlighted the importance of 11-oxygenated C19 steroids to the androgen pool in humans. The aim of this study was to confirm the origin of 11-oxygenated androgens in females and to explore their potential use in the diagnostics of hyperandrogenic disorders.

**Methods:**

We measured testosterone and its precursors (dehydroepiandrosterone-sulfate and androstenedione) and 11-oxygenated androgens (11β-hydroxyandrostenedione (11-OHA4) and 11-ketotestosterone (11-KT)) in the periphery, adrenal and ovarian veins in four different cases of hyperandrogenism in females (polycystic ovary syndrome (PCOS), primary bilateral macronodular adrenal hyperplasia, Sertoli–Leydig cell tumor and ovarian steroid cell tumor).

**Results:**

Two patients demonstrate excessive testosterone secretion in neoplastic ovarian tumors which was not paralleled by a significant secretion of 11-oxygenated androgens as determined by adrenal and ovarian vein sampling. In androgen-secreting bilateral adrenal macronodular hyperplasia, steroid profiles were characterized by elevated 11-KT and 11-OHA4 concentrations in adrenal veins and the periphery. In the patient with PCOS, peripheral 11-KT concentrations were slightly elevated in comparison to the other patients, but the 11-KT and 11-OHA4 concentrations were comparable in ovarian veins and in the periphery.

**Conclusion:**

This study confirms that 11-OHA4 and 11-KT are not biosynthesized by the ovary. We propose that the testosterone/11-KT ratio as well as 11-OHA4 could help identify predominant adrenal androgen excess and distinguish neoplastic and non-neoplastic ovarian androgen source.

**Significance statement:**

This study confirms that 11β-hydroxyandrostenedione (11-OHA4) and 11-ketotestosterone (11-KT) are not biosynthesized by the human ovary. We propose that the testosterone/11-KT ratio as well as 11-OHA4 could help to identify predominant adrenal androgen excess and distinguish neoplastic and non-neoplastic ovarian androgen source.

## Introduction

Disorders of androgen excess are common in the general female population affecting up to 15% of women (1). Most of these women suffer from polycystic ovary syndrome (PCOS) (2) or non-classic congenital adrenal hyperplasia (CAH) (3). Underlying androgen-secreting tumors are rare and only account for about 0.2–1.7% of all cases.

In case of suspected neoplastic androgen secretion, differentiation between an adrenal or ovarian origin can be challenging and require suppression testing (4, 5, 6, 7, 8, 9), extensive imaging diagnostics (10, 11) and invasive procedures such as selective venous sampling (4).

**Figure 1:**
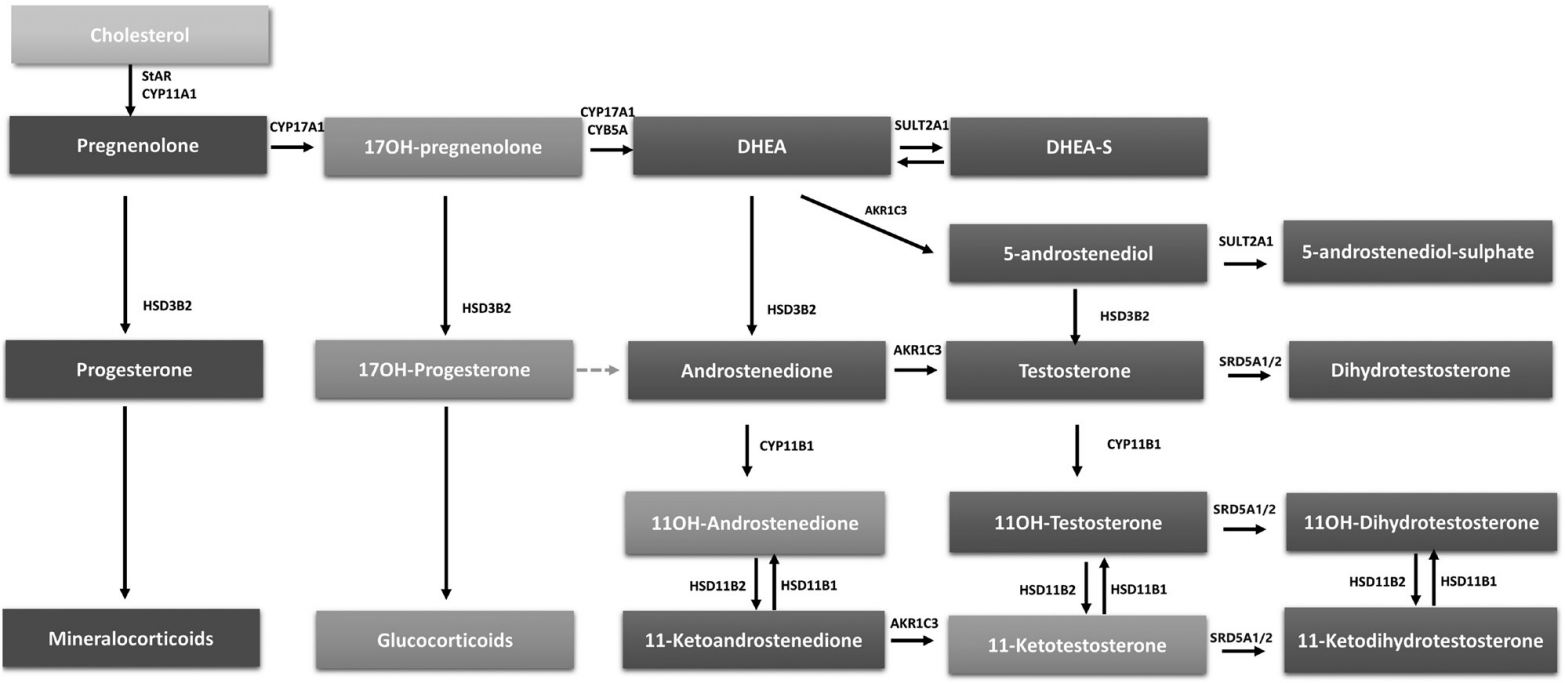
Biosynthesis of 11-oxygenated androgens.

Even though 11-oxygenated 19-carbon steroids have been known for decades, they have recently gained increasing attention because of their contribution to several hyperandrogenic disorders (12, 13, 14). The term collectively refers to the 11β-hydroxyandrostenedione (11-OHA4), 11β-hydroxytestosterone (11-OHT), 11-ketoandrostenedione (11-KA4) and 11-ketotestosterone (11-KT) steroids. Among these, it has been demonstrated that 11-KT and its derivate 11-KT-dihydrotestosterone act at the androgen receptor with equal potency to the classic androgens testosterone and dihydrotestosterone, respectively (15) highlighting their clinical significance (14). Androstenedione (A4) is converted to 11-OHA4 by cytochrome P450 11β-hydroxylase (CYP11B1), then to 11-KA4 through the action of 11β-hydroxysteroid dehydrogenase type 2 and finally to 11-KT by Aldo-keto reductase family 1 member C3 (AKR1C3) (16) (Fig. 1). In contrast to 11-OHA4 and 11-OHT, 11-KT and 11-KA4 are considered to be mainly produced in peripheral tissues (16). As the biosynthesis of 11-oxygenated androgens depends on cytochrome P450 family 11 subfamily B member 1 (CYP11B1), an adrenal-specific enzyme, they should also be adrenal-specific (13). It has been shown that 11-oxygenated androgens are elevated in adrenal-related androgen excess as CAH or premature adrenarche and Cushing’s disease (14, 17, 18). In addition, they may also be elevated in some (19, 20), but not all patients (21) with PCOS. However, as there is also* in-vitro* data claiming that there is a relevant production of 11-KT derived from the gonads (15), details of the biosynthesis of 11-oxygenated androgens in humans are debatable as direct evidence in terms of* in-vivo* data of localized secretion patterns is absent. If 11-oxygenated androgens are highly specific for adrenal androgen secretion, it would support their utility in the diagnostic workup of hyperandrogenism in women.

By presenting four cases of women with rare or challenging causes of androgen excess who had undergone an extensive diagnostic workup including peripheral as well as adrenal vein and ovarian vein blood sampling, our aim was to confirm the source of 11-oxygenated androgens production and to explore if their peripheral measurement could benefit the differential diagnostic workup of cases with an unclear source of androgen excess.

## Methods

### Subjects

All patients were seen at the outpatient clinic of the Medizinische Klinik IV, Klinikum der Universität München, LMU Munich. Written informed consent was obtained from the individuals described in this case series and all patients participated in our patient registries and biobanks that were approved by the Ethical review board of the Ludwig Maximilian University, Munich (Bio AI/DSD, ethical approval no. 19-558, NeoExNET, ethical approval no. 152-10).

### Hormonal assays

Adrenocorticotropic hormone (ACTH), luteinizing hormone, follicle-stimulating hormone, β-human chorionic gonadotropin, estradiol, cortisol and dehydroepiandrosterone sulfated metabolite (DHEAS) measurements were determined using the Liaison chemiluminescence immunoassays (DiaSorin, Sallugia, Italy). Total testosterone, 17-OHP, A4, 11-KT and 11-OHA4 were quantified by LC-MS/MS. Steroid extraction and quantitation were conducted as previously described (22). Serum 11-KT and 11-OHA4 were determined in preserved samples retrospectively. In the case of selective venous sampling, the presented results refer to the mean values of two to three successful samplings per site. For adrenal venous sampling, mean values of both sides are presented, as there was no significant lateralization in the presented cases.

## Results

### Case descriptions

**Figure 2:**
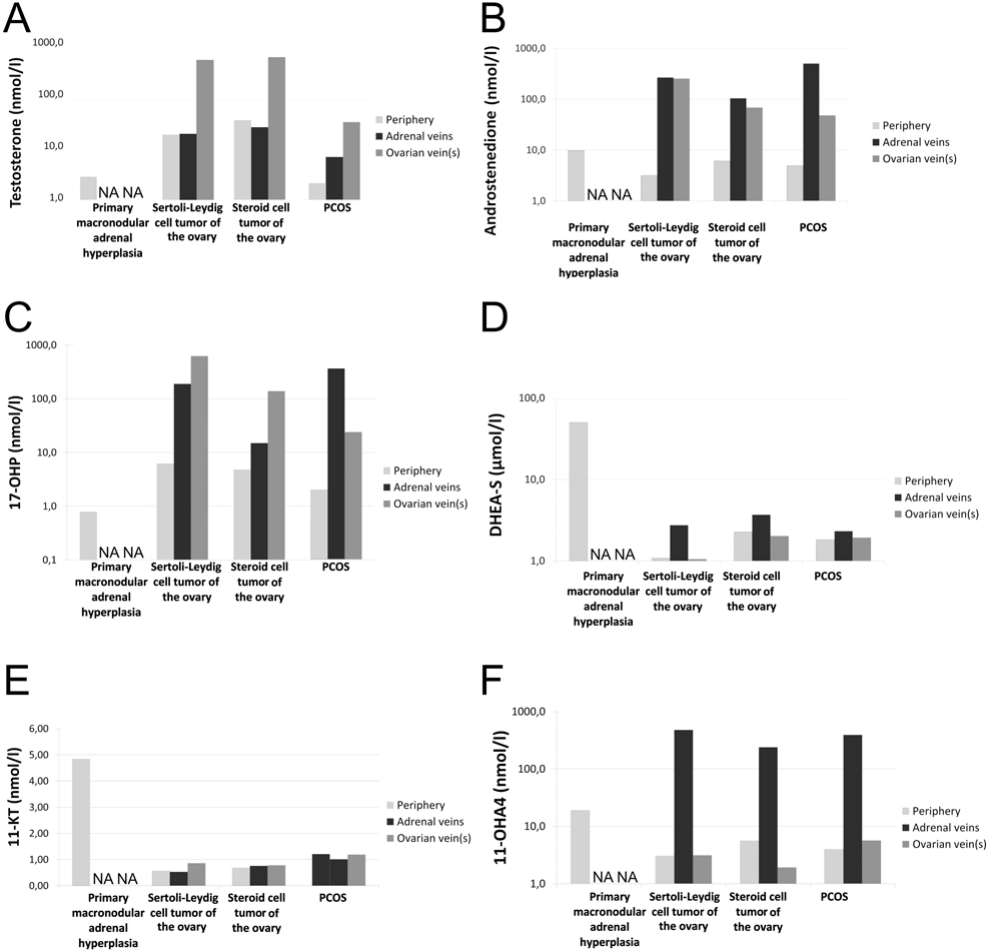
Steroid levels at different sampling sites. NA, not available; 11-KT, 11-ketotestosterone; 11-OHA4, 11β-hydroxyandrostenedione; 17-OHP, 17-hydroxyprogesterone. In the case of selective venous sampling, the presented results refer to the mean values of two to three successful samplings per site. For adrenal venous sampling, mean values of both sides are presented, as there was no significant lateralization in the presented cases.

**Figure 3:**
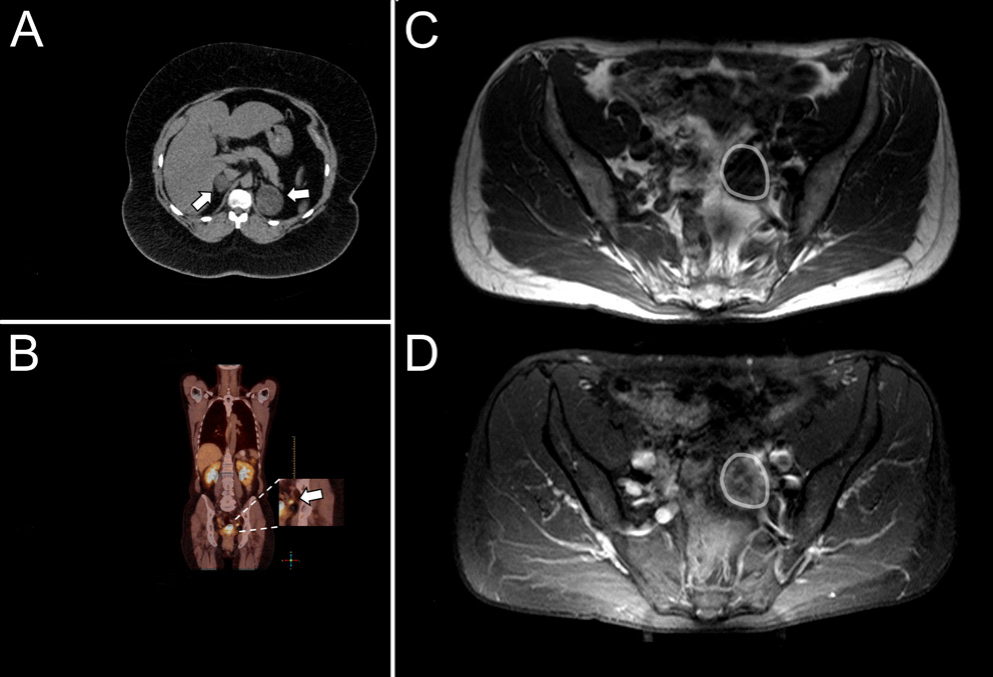
Computed tomographic scan showing bilateral macronodular adrenal hyperplasia (Patient 1, A); PET-CT revealing a left-sided ovarian mass (Patient 2, B); MRI scan revealing a cystic tumor on the left ovary (C) with peripheral contrast media enhancement (Patient 3, D).

Case 1 (macronodular adrenal hyperplasia) was a 45-year-old woman who had suffered from secondary amenorrhea since the age of 33. She was further suffering from poorly controlled diabetes, hirsutism, presented with cushingoid stigmata and was severely obese (BMI 43.9 kg/m²). Moderate hyperandrogenemia was diagnosed with a total serum testosterone of 2.9 nmol/L (0.31–2.29 nmol/L) (Fig. 2, Supplementary Table 1, see section on supplementary materials given at the end of this article). Cortisol suppression following 1 mg dexamethasone was insufficient (6.6 µg/dL) and suppressed ACTH levels indicated an adrenal source of glucocorticoid- and androgen-excess (Supplementary Table 1). A CT scan revealed bilateral (macronodular) adrenal hyperplasia (Fig. 3A), and a gynecological examination provided no evidence of an ovarian tumor. Due to the larger left nodule, the patient was referred for unilateral adrenalectomy.

Case 2 (Sertoli-Leydig cell tumor) was a 72-year-old woman presenting with significant hirsutism and male-pattern hair loss starting which started approximately 3 months before referral to our endocrine outpatient unit. She had a history of unilateral ovariectomy and hysterectomy due to endometriosis. Total serum testosterone concentration was extremely elevated at 19.7 nmol/L (0.31–2.29 nmol/L). 17-OHP was also elevated, while DHEAS and A4 were within their respective age- and sex-specific reference ranges. There was an adequate cortisol suppression following the intake of 1 mg dexamethasone while there was no significant suppression of testosterone or DHEAS (Supplementary Table 1). An abdominal CT scan revealed nodular hyperplasia of the right adrenal (1 cm), while there was no evidence of a tumor in the remaining left ovary. In a subsequent PET-CT scan, there was significant tracer accumulation in the left ovary (Fig. 3B). Simultaneous selective adrenal and ovarian venous sampling showed that testosterone concentrations from the adrenal veins were comparable to those sampled from the periphery while conversely, testosterone concentration from the remaining left ovarian vein was 31× greater than that obtained from the periphery (Fig. 2).

Case 3 (steroid-cell tumor) was a 54-year-old woman suffering from male-pattern hair loss and hirsutism that began 3 to 4 years ago. She had been postmenopausal for 2 years and had recognized deepening of the voice over the same period as well as clitoral hypertrophy. Total serum testosterone concentrations were extremely elevated at 38 nmol/L, while A4, 17-OHP and DHEAS were only slightly increased (Supplementary Table 1). An MRI scan of the abdomen revealed a 3 cm cystic tumor on the left ovary while there was no sign of any adrenal tumor (Fig. 3C/D). Selective adrenal and ovarian venous sampling showed that testosterone from the left ovary was 18× greater than in the peripheral sample and the healthy right ovary (21×). A4 (11.2× periphery, 17.5× right ovary) and 17-OHP (29× periphery, 37× right ovary) were also significantly elevated (Fig. 2, Supplementary Figure 1). In contrast, total testosterone concentrations measured from the adrenal veins were lower than from the periphery. The patient was referred for laparoscopy and the pathological examination revealed a steroid-cell tumor as the underlying cause of androgen excess.

Case 4 (PCOS) was a severely obese (BMI 40.4 kg/m²) 42-year-old female patient suffering from moderate hirsutism for approximately 5 years. She was further presenting with secondary amenorrhea and arterial hypertension. She had no classic cushingoid stigmata, but severe insulin resistance was suspected due to the presence of pronounced acanthosis nigricans. She had undergone unilateral ovariectomy several years prior due to several benign cystic lesions of the left ovary (in retrospective in accordance with PCOS). Total serum testosterone was borderline high (2.1 nmol/L), while A4, DHEAS and 17-OHP were within the female reference range. Given that the patient had been amenorrheic for 2 years, estradiol levels were high (269 pg/mL). Cortisol excess was excluded (Supplementary Table 1). CT scanning did not detect any lesions. A pelvic ultrasound showed that the ovarian volume of the remaining ovary was increased and included multiple follicles up to 11 mm. Selective adrenal and ovarian venous sampling revealed that testosterone concentrations in the adrenal vein were only slightly elevated in comparison to the periphery (left: 3.4×; right 2.4×) but significantly elevated in the remaining left ovarian vein (16.6×) (Fig. 2).

### 11-Oxygenated androgens

In all cases, 11-OHA4 levels in the adrenal veins were significantly higher than in the ovarian veins (Fig. 2). Conversely ovarian concentrations were either comparable to (Cases 2 and 4) or even lower than those obtained from the periphery (Case 3). Concentrations of 11-KT were not higher in the ovarian veins compared to the periphery in any of the cases with ovarian androgen excess. In Case 3, 11-KT derived from the healthy right ovary was not higher than in the periphery (0.5 vs 0.7 nmol/L; Supplementary Fig. 1). Systemic concentrations of 11-KT in the two patients with ovarian androgen excess were low in comparison to the patient with adrenal androgen excess due to bilateral macronodular adrenal hyperplasia (Fig. 3; Supplementary Fig. 1). In PCOS, there was no significant difference in 11-KT levels obtained from the different sampling sites (ovarian veins: 1.2 nmol/L: adrenal veins: 1.0 nmol/L, periphery: 1.2nmol/L). The 11-KT levels were about 2× times higher in the periphery compared to the 2 patients with neoplastic hyperandrogenism (Case 2: 0.6 nmol/L, Case 3: 0.7 nmol/L) (Fig. 2).

## Discussion

To the best of our knowledge, this is the first study reporting * in-vivo* data from ovarian vein samplings for 11-KT and 11-OHA4 concentrations.

In all the study cases, adrenal and ovarian venous sampling confirmed that 11-OHA4 is secreted abundantly from the adrenal glands (12). In contrast, concentrations of 11-KT were not higher in the ovarian veins compared to the periphery in any of the patients with ovarian androgen excess. This finding confutes the results of a previous study that claims that 11-KT represents a major androgen produced in the human gonads* in-vivo* (15). Although it has been shown that 11β-hydroxysteroid dehydrogenase types 1 and 2 are expressed in testicular Leydig and ovarian theca cells (15), our findings contradict the idea that gonadal 11-KT production significantly contributes to the systemic pool in females because *CYP11B1* is usually only expressed in negligible amounts in the gonads (23). An exception to this are patients with CAH in which aberrant adrenal tissue can be found within the testicles or ovaries (24, 25). The idea that 11-KT is produced in peripheral tissue from 11-OHA4 produced in the adrenal is supported by the observation that 11-KT levels did not differ between the sampling sites, considering the significantly higher 11-OHA4 levels in the adrenal vein samples, especially. Peripheral 11-KT concentrations were also considerably higher in the patient with macronodular hyperplasia in comparison to the other patients. As selective venous sampling had not been performed in this patient due to the unequivocal imaging, we can only speculate on its major source, but it is likely to have been derived from the peripheral conversion of excessive amounts of 11-OHA4. As this patient was severely obese due to concomitant cortisol excess, it is conceivable that increased 11-KT secretion was derived from excessive adipose tissue conversion of 11-OHA4 and 11-KA4 by AKR1C3 that can be further stimulated by hyperinsulinemia (26).

In the patient with PCOS, peripheral 11-KT levels were only slightly higher than those in the two patients with tumorous ovarian androgen secretion. There was no difference in 11-KT levels obtained from the different sampling sites, again challenging the idea of a primarily ovarian 11-KT biosynthesis. While all published studies on PCOS and 11-oxygenated androgens (19, 20, 27) showed elevated levels of 11-OHA4 in comparison to controls, 11-KT seems only to be elevated in some (19, 20), but not all women with PCOS (19, 27). An elevation of 11-KT seems to be primarily found in women with PCOS who are obese but not in those who are only over- or normal-weighted (19, 20). This may be due to AKR1C3 activity in adipose tissue (26). These discrepancies may also be explained by the fact that PCOS encompasses a wide variety of disease patterns (2), among other factors being defined by the degree of ovarian and adrenal androgen secretion (28, 29).

### Limitations

A limitation of our study is the presentation of single cases that do not allow for the generalization of the results. Additionally, we did not measure 11-KA4 and 11-OHT levels. 11-OHT might have been a better marker to determine if ovarian tumors were able to directly 11βhydroxylate testosterone to 11-OHT, especially as testosterone was more abundant than androstenedione in the ovarian vein samples. However, the quantification of these steroids is hampered by the fact that there are no deuterated internal standards currently available. Finally, we did not perform selective venous sampling in the patients with macronodular hyperplasia, given the obvious adrenal origin of androgen excess.

Nonetheless, although our cases are anecdotal, we find them encouraging regarding the potential utility of peripheral 11-oxygenated androgen measurements for improving the differential diagnosis in severe hirsutism and/or hyperandrogenemia. Our data support the assumption that 11-KT concentrations are low relative to the high concentrations of circulating testosterone which are typical of androgen-secreting ovarian tumors. The data are particularly encouraging as two different entities of these ovarian tumors were examined in our study, providing similar results. A testosterone/11-KT ratio may therefore be a promising variable to differentiate neoplastic ovarian from non-neoplastic ovarian sources such as PCOS, while 11-OHA4 may further help to differentiate between adrenal and ovarian tumors.

## Conclusion

Our data clearly illustrate and give direct evidence that 11-oxygenated androgens are of adrenal origin and not produced in the human ovary as claimed previously (15). Based on our findings, we hypothesize that a serum testosterone/11-KT ratio has the potential for differentiating neoplastic from non-neoplastic ovarian androgen excess. Furthermore, measurement of serum 11-OHA4 may help delineate ovarian from adrenal androgen secretion. Larger studies are required to further investigate the diagnostic performance of 11-KT and 11-OHA4 in larger cohorts of rare cases of hyperandrogenism.

## Supplementary Material

Supplementary figure S1: Steroid levels in case 3 at different sampling sites

Supplementary table S1 Peripheral hormone levels

## Declaration of interests

The authors report no conflicts of interest in this work. Nicole Reisch is on the editorial board of the *European Journal of Endocrinology*. Nicole Reisch was not involved in the review or editorial process for this paper, on which he/she is listed as an author.

## Funding

This work was supported by the IFCAH grant 2013 to NR and by the Deutsche Forschungsgemeinschaft http://dx.doi.org/10.13039/501100001659 (Heisenberg Professorship 325768017 to NR and Projektnummer: 314061271-TRR205 to NR).
