# Supplementary figures and images for: 11-Oxygenated androgens are not secreted by the human ovary: in-vivo data from four different cases of hyperandrogenism
Source: Eur J Endocrinol. 2022 Oct 13;187(6):K47–53. doi: 10.1530/EJE-22-0518 (PMC9716487; doi:10.1530/EJE-22-0518)

### Case 3 (steroid-cell tumor)

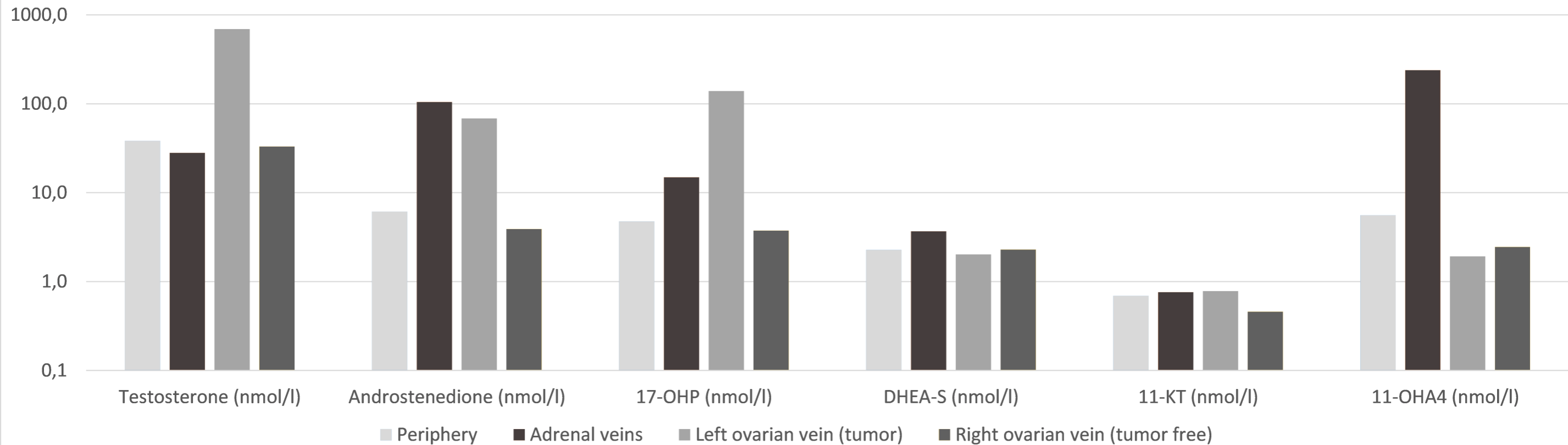

Supplement: Supplementary figure S1: Steroid levels in case 3 at different sampling sites [file supplementary_figure_1.pdf]
