# Supplementary material for: 11-Oxygenated androgens are not secreted by the human ovary: in-vivo data from four different cases of hyperandrogenism
Source: Eur J Endocrinol. 2022 Oct 13;187(6):K47–53. doi: 10.1530/EJE-22-0518 (PMC9716487; doi:10.1530/EJE-22-0518)
Supplement: Supplementary table S1 Peripheral hormone levels [file supplementary_table_1.pdf]

Table 1 Peripheral hormone levels

**Hormone**	**Patient 1 (Macronodular hyperplasia)**	**Patient 2 (Sertoli-Leydig-Cell-Tumor)**	**Patient 3 (steroid-cell tumor)**	**Patient 4 (PCOS)**	**Reference range**
Testosterone (nmol/l)	**2.9**	**19.7**	**38.2**	2.1	0.31–2.29[1]
17-OHP (nmol/l)	0.8	6.2	4.8	2	0.24–6.84(40)[1]
DHEA-S (µmol/l)	**50.2**	1.1	2.3	1.8	8.5–7.44(40)[1]
Androstenedione (nmol/l)	**9.8**	3.2	6.1	5	1.06–7.72(40)[1]
11-KT (nmol/l)	**4.84**	0.57	0.7	1.2	0.46-1.26[2]
11-OHA4 (nmol/l)	**19**	3.1	5.6	4	1.92-6.95[2]
T/11-KT-ratio	0.6	34.6	54.6	1.75	NA
ACTH (pg/ml)	**<2**	8	14	16	4-50
Prolactin (µU/ml)	**7931**	165	NA	156	<496
LH (U/l)	<0.1	19.7	NA	4.9	Age- and cycle-dependent
FSH (U/l)	0.4	18.7	NA	3.7	Age- and cycle-dependent
Estradiol (pg/ml)	43.1	103	NA	269	Age- and cycle-dependent
Cortisol following 1mg dexamethasone (µg/dl)	**6.6**	1.5	1.5	0.8	<2
β-HCG (ug/l)	NA	0.2	<0.1	NA	< 0.5

NA: not available, 11-KT: 11-ketotestosteron, 11-OHA4: 11β-hydroxyandrostenedione, DHEA-S: dehydroepiandrosterone sulfate 17-OHP: 17-hydroxyprogesterone

References

1. Eisenhofer, G., et al., *Reference intervals for plasma concentrations of adrenal steroids measured by LC-MS/MS: Impact of gender, age, oral contraceptives, body mass index and blood pressure status.* Clin Chim Acta, 2017. **470**: p. 115-124.
2. Nanba, A.T., et al., *11-Oxygenated C19 Steroids Do Not Decline With Age in Women.* J Clin Endocrinol Metab, 2019. **104**(7): p. 2615-2622.
